# Enhancing Selectivity and Inhibitory Effects of Chemotherapy Drugs Against Myelogenous Leukemia Cells with *Lippia alba* Essential Oil Enriched in Citral

**DOI:** 10.3390/ijms25168920

**Published:** 2024-08-16

**Authors:** Wendy Lorena Quintero-García, Denerieth Ximena Espinel-Mesa, Erika Marcela Moreno, Elena Stashenko, Ana Cecilia Mesa-Arango, Liliana Torcoroma García

**Affiliations:** 1 Corporación Académica Ciencias Básicas Biomédicas, Universidad de Antioquia, Medellín 050010, Colombia; wendy.quinterog@udea.edu.co (W.L.Q.-G.); den.espinel@mail.udes.edu.co (D.X.E.-M.); ana.mesa@udea.edu.co (A.C.M.-A.); 2 Facultad de Ciencias Médicas y de la Salud, Departamento de Postgrado en Enfermedades Infecciosas, Universidad de Santander, Bucaramanga 680006, Colombia; coord.maestriainfecciosas@udes.edu.co; 3 National Research Center for the Agroindustrialization of Aromatic and Medicinal Tropical Species (CENIVAM), Universidad Industrial de Santander, Bucaramanga 680002, Colombia; elena@tucan.uis.edu.co

**Keywords:** acute myelogenous leukemia, citral, chemotherapy, *Lippia alba*

## Abstract

Acute myelogenous leukemia (AML) is one of the most lethal cancers, lacking a definitive curative therapy due to essential constraints related to the toxicity and efficacy of conventional treatments. This study explores the co-adjuvant potential of *Lippia alba* essential oils (EO) for enhancing the effectiveness and selectivity of two chemotherapy agents (cytarabine and clofarabine) against AML cells. EO derived from *L. alba* citral chemotype were produced using optimized and standardized environmental and extraction protocols. Rational fractionation techniques were employed to yield bioactive terpene-enriched fractions, guided by relative chemical composition and cytotoxic analysis. Pharmacological interactions were established between these fractions and cytarabine and clofarabine. The study comprehensively evaluated the cytotoxic, genotoxic, oxidative stress, and cell death phenotypes induced by therapies across AML (DA-3ER/GM/EVI1+) cells. The fraction rich in citral (F2) exhibited synergistic pharmacological interactions with the studied chemotherapies, intensifying their selective cytotoxic, genotoxic, and pro-oxidant effects. This shift favored transitioning from necrosis to a programmed cell death phenotype (apoptotic). The F2-clofarabine combination demonstrated remarkable synergistic anti-leukemic performance while preserving cell integrity in healthy cells. The observed selective antiproliferative effects may be attributed to the potential dual prooxidant/antioxidant behavior of citral in *L. alba* EO.

## 1. Introduction

Acute myelogenous leukemia (AML) is a heterogeneous and aggressive neoplasm, representing the second leading cause of cancer-related death in children and adolescents aged 2–19 years old [1]. AML progression and mortality risks are primarily associated with delays in diagnosis and treatment, poor health conditions that prevent standard intensive treatment, multi-drug resistance infections, and various socio-economic barriers [2]. Currently, to achieve rapid remission and control leukocytosis, a 7-day continuous infusion of cytarabine (100 or 200 mg/m^2^/day IV), combined with anthracycline treatment (12 mg/m^2^/day IV) on days 1 and 3, is used as induction chemotherapy (the “7 + 3” regimen). However, this regimen can require adjustments in patients who, due to their age or weakness, cannot tolerate it because of its high toxicity and low selectivity [3,4]. Despite intensive conventional chemotherapy for AML, complete remission is achieved only in 60 to 80% of younger adults and 40 to 60% of older individuals (aged >60 years) [5], with approximately 50% of cases experiencing cancer recurrence. Additionally, treatment-related mortality in AML is significant, with rates ranging from 19% for patients under 65 years old [3] to 14% for older individuals [4]. Consequently, a significant proportion of patients succumb to this disease, with a five-year overall survival rate of less than 60% in those younger than 14 years old and less than 15% in persons aged 65 years or older [6].

The significant constraints caused by the non-specific cytotoxicity of chemotherapy lead to severe toxicity, inflammatory responses, and systemic immunosuppression. Consequently, clinicians may need to prescribe lower doses, which increase the risks of inactivation and resistance, or resort to higher, toxic doses that increase the risk of a second neoplasm, high toxicity in healthy organs, and death [7]. Novel strategies should address leukemia’s complex pathogenesis and chemotherapy’s limitations. Due to their mechanisms of action on multiple cellular targets, including potentiation of programmed cell death (PCD) mechanisms, inhibition of proliferative pathways, and modulation of molecules and cells involved in persistent inflammatory and oxidative states, phytoderivatives appear to be feasible complementary approaches to conventional cytotoxic drugs.

Plant-extracted terpenes or their synthetic derivatives (such as paclitaxel and docetaxel) have become established standard therapies for prostate, lung, ovarian, and breast cancer [8,9]. Additionally, selective and broad-spectrum anti-tumoral properties have been described from preclinical and clinical studies for terpenes like citral [10,11,12]. These properties are associated with the selective induction of oxidative stress in tumor cells by increasing reactive oxygen species (ROS) through their reaction with intracellular nucleophiles and depleting reduced glutathione (GSH) tab levels [10]. Consequently, citral triggers intrinsic apoptosis pathways through p53 phosphorylation, upregulation of Bax, downregulation of Bcl-2 and Bcl-xL, mitochondrial membrane depolarization, externalization of phosphatidylserine (PS), caspase-3 cleavage, and DNA degradation [11,13]. Furthermore, it induces cell cycle arrest at the G2/M phase [14]. Remarkably, these cytotoxic effects of citral are highly selective for tumoral cells, with minimal antiproliferative or harmful actions on healthy cells [11]. In non-tumoral cells, this terpene exhibits immunomodulatory, anti-inflammatory, antioxidant, and cytoprotective properties [15,16]. The antioxidant behavior of citral is attributed to its C-H aldehyde reaction with oxygen radicals [17]. Consistently, citral is a GRAS (generally recognized as safe) compound extensively employed as a flavor additive in foods, beverages, and perfumes [18].

Oxidative stress has emerged as a promising mechanism for selectively inducing tumor cell death. This phenomenon is based on the significant difference in the basal levels of ROS between normal cells and malignant cells, with normal cells having much lower levels [19]. This difference is influenced by the hypoxic environment, the high metabolic activity of tumor cells due to their constant proliferation, and the inferior performance of their antioxidant defense systems [20]. This study hypothesizes that because tumor cells survive at the threshold of elevated ROS levels, inducing oxidative stress can potentially lead to their death [19]. Therefore, using dual-nature agents (prooxidants/antioxidants) that selectively induce oxidative stress in malignant tissue beyond their tolerance while acting as cytoprotectants (antioxidants) on healthy cells holds promise when combined with chemotherapeutic agents [20].

This study aims to enhance the efficacy of chemotherapeutics (cytarabine and clofarabine) while minimizing nonspecific cytotoxicity in non-tumoral cells. To achieve this, the cytotoxic and antiproliferative properties of *Lippia alba* (Miller) N.E. Brown (Verbenaceae) essential oils (EO) isolated from the citral chemotypes were assessed on AML (DA-3ER/GM/EVI1+) and healthy (Vero and J774A.1) cells. *L. alba* is an aromatic shrub that grows wild in the Colombian Andean Mountains [11]. For reproducibility, EO were obtained under standardized agricultural and production parameters [11]. Additionally, the oils were fractionated to obtain terpene-enrichment fractions guided by correlation analysis of bioactivity (antiproliferative and selective effects) relative to the chemical composition obtained by Gas Chromatography-Mass Spectrometry (GC-MS). Pharmacological interactions between EO and fractions with cytarabine and clofarabine were explored. The cell death phenotype induced by the treatments was assessed through cell morphology, mitochondrial membrane potential, oxidative stress, antioxidant defense systems, caspase activation, and PS externalization assays.

## 2. Results

### 2.1. Chemical Composition of L. alba EO and Fractions

Table 1 presents the relative chemical composition and the linear retention indices obtained through Gas Chromatography-Mass Spectrometry (GC-MS) analysis of the five EO (EOCit1 to EOCit5) and the citral-rich fraction (F2) isolated from the citral chemotypes of *L. alba* under standardized (planting, collecting, and extraction of vegetal material) conditions. Neral and geranial terpenes, the primary constituents of citral, were identified as the major compounds in oils and the F2 fraction.

### 2.2. Cytotoxic Activity of L. alba EO and Its Fraction

**Figure 1:**
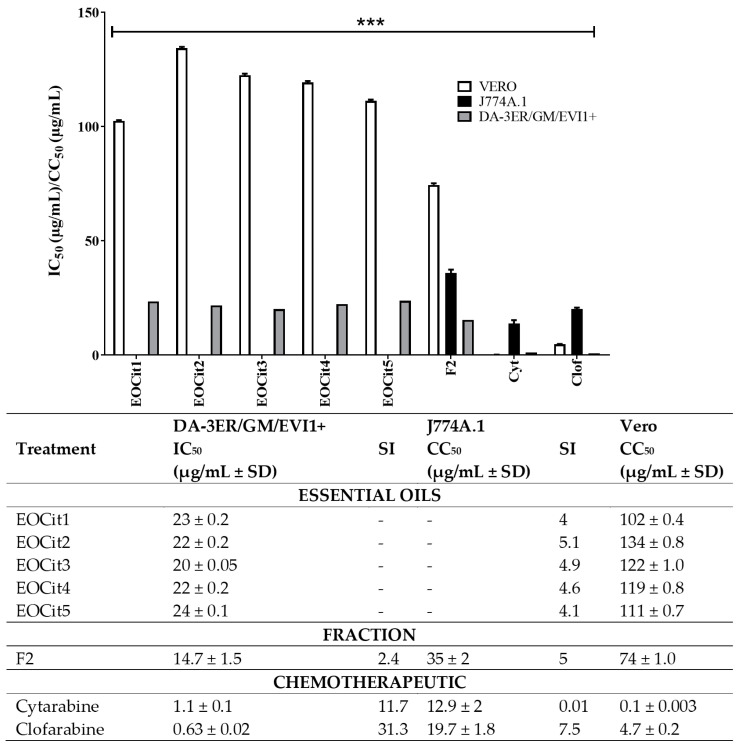
Antiproliferative and cytotoxic activity. EOCit1-EOCit5: EO from citral chemotype of *L. alba*; F2: citral-rich fraction; IC_50_: inhibitory concentration 50; CC_50_: cytotoxic concentration 50; SD: standard deviation; SI: selectivity index (CC_50_/IC_50_), ***: *p* < 0.0001.

All the EO extracted from the citral chemotype of *L. alba* (EOCit1 to EOCit5) exhibited significant anti-proliferative activity (*p* < 0.0001) against AML (DA-3ER/GM/EVI1+) cells. However, EOCit2 demonstrated the highest anti-tumoral and selective performance (Figure 1). Based on these results, further rational fractioning using reduced-pressure fractional distillation of the citral chemotype EO resulted in a fraction enriched in citral terpene (F2). This fraction was predominantly composed of citral, comprising 72%, as a racemic mixture of neral (~40%) and geranial (~32%). In antiproliferative assays, F2 exhibited significant inhibitory effects on the growth of the tumor cell line (Figure 1). Additionally, F2 maintained low cytotoxicity in Vero and J774A.1 cells (Figure 1).

### 2.3. Pharmacological Interactions between Terpenes and Chemotherapeutics

**Figure 2:**
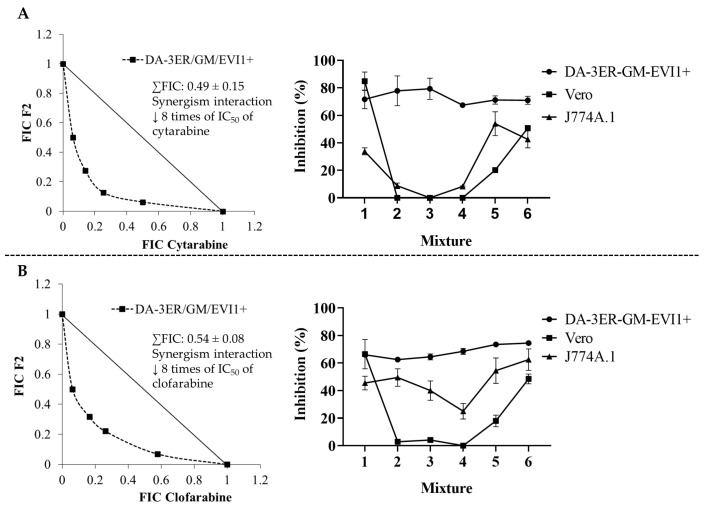
Isobolograms of pharmacological interactions and inhibition percentages between F2 and chemotherapies. Cytarabine (**A**); Clofarabine (**B**). FIC: fractional inhibitory concentration; F2: citral-rich fraction, ↓: decrease.

Because F2 demonstrated the most effective selective inhibitory effects on AML cells, this fraction was selected as the fixed compound for the pharmacological interaction matrix with cytarabine or clofarabine. This matrix was constructed using each compound’s previously defined inhibitory concentration 50 (IC_50_) values (Figure 1). The drug interaction data were visualized as graphs through isobolograms, plotting each combination’s mean Fractional Inhibitory Concentration (FIC) (Figure 2). On DA-3ER/GM/EVI1+ cells, the highest antiproliferative performance was demonstrated by combined therapies using the IC_50_ of chemotherapies plus twice the IC_50_ of F2 [1X (IC_50_) cytarabine/clofarabine + 2X (IC_50_) F2]. This mixture reduced the individual IC_50_ values by 8-fold for both drugs. Notably, a cytoprotective effect against the toxic impact of these drugs on non-tumoral cells was observed when F2 was added to cytarabine or clofarabine. Specifically, growth cell inhibition was undetectable in Vero cells, and minimal in murine macrophages (8% with cytarabine and 21% with clofarabine) after 24 h of treatment (Figure 2).

### 2.4. Morphological Changes and Oxidative Stress Triggered by Treatments

**Figure 3:**
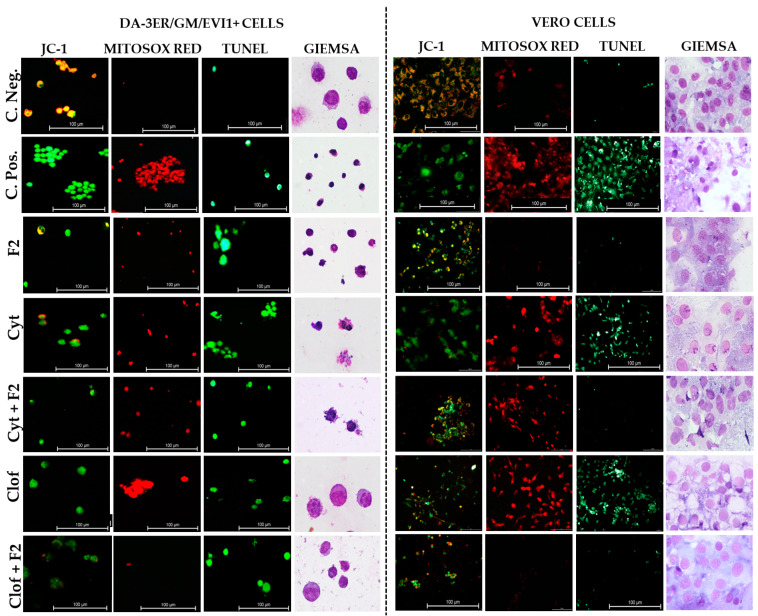
Morphology of cells treated with the studied therapies. Cyt: cytarabine; Clof: clofarabine; F2: citral-rich fraction. IC_50_: Inhibitory Concentration 50; CC_50_: Cytotoxic Concentration 50; C. Pos: cells treated with carbonyl cyanide 3-chlorophenylhydrazone; C. Neg: untreated cells.

AML cells (DA-3ER/GM/EVI1+) treated with cytarabine displayed diminished cytoplasmic volume, karyopyknosis (as observed with the Giemsa stain), partial loss of mitochondrial membrane potential (depletion of yellow-stain cells marked with the JC-1 probe), and DNA fragmentation (TUNEL-positive cells). This cytotoxic effect was non-selective since Vero cells presented detachment, total loss of mitochondrial membrane potential (green-stained cells in JC-1) and reduced cytoplasmic volume (Figure 3). Interestingly, when F2 was added to cytarabine, a significant reduction in the detrimental impact of this drug on Vero cells was observed, resulting in the restoration of cell adhesion and mitochondrial membrane potential (Figure 3).

On the other hand, DA-3ER/GM/EVI1+ and Vero cells treated with clofarabine exhibited cell edema and loss of cell membrane integrity. However, combined therapy of this drug with F2 shifted the morphology from necrotic to programmed cell death (PCD) morphology in AML cells. For instance, DA-3ER/GM/EVI1+ cells treated with F2-clofarabine displayed reduced cytoplasmic volume, chromatin condensation, nuclear fragmentation, and loss of mitochondrial membrane potential, suggesting the activation of a possible apoptotic cell death mechanism. In contrast, adding F2 to clofarabine exhibited a cytoprotective effect in non-tumoral cells. Consequently, Vero cells treated with this combined therapy demonstrated a significant decrease in mitochondrial oxidative stress (reduction in levels of MitoSOX™ Red fluorescence), preservation of DNA integrity (TUNEL negative), cell adherence, and maintained mitochondrial membrane potential (orange and yellow stain in cells) (Figure 3).

### 2.5. Genotoxicity on Tumoral and Vero Cells

**Figure 4:**
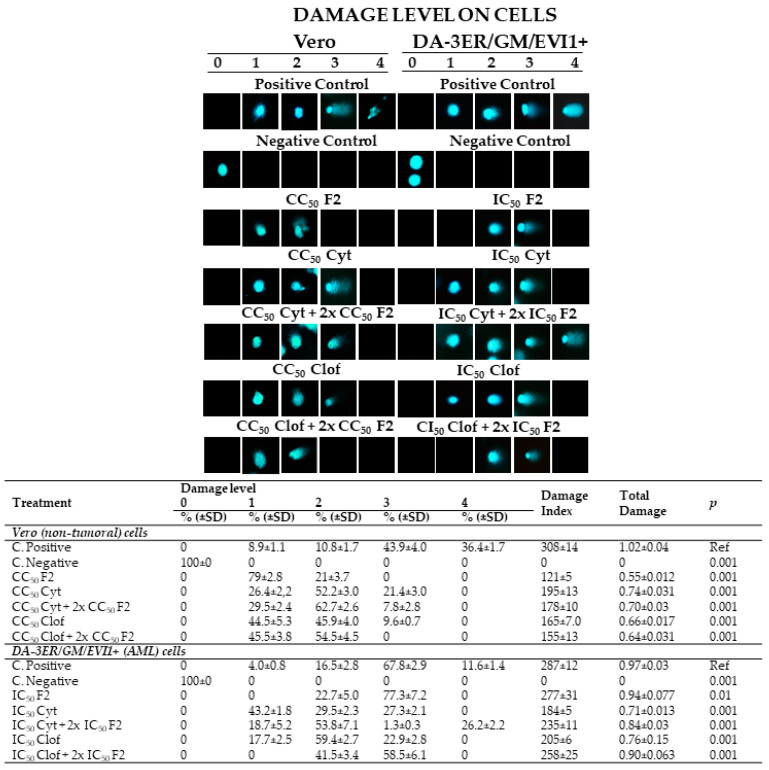
Genotoxicity by comet assay. Cyt: cytarabine; Clof: clofarabine; F2: citral-rich fraction; IC_50_: inhibitory concentration 50; CC_50_: cytotoxic concentration 50.

On DA-3ER/GM/EVI1+ cells, cytarabine exhibited moderate genotoxicity, causing type 1 damage (at a lower level) in approximately 50% of the cells. However, when combined with the citral-enriched fraction (F2), this genotoxic action significantly intensified in leukemia cells. For instance, while AML cells treated with cytarabine alone showed 0% maximal damage to the genome (type 4), adding F2 triggered 26.2% of this type of genotoxicity. Conversely, in Vero cells, F2 with cytarabine mitigated the DNA damage caused by this chemotherapy, reducing type 3 damage threefold (from 21.4 ± 3.0 to 7.8 ± 2.8) and resulting in minimal injury in 92% of the treated cells (damage level types 1 and 2) (Figure 4).

On DA-3ER/GM/EVI1+ cells, the combination of clofarabine with F2 potentiated the genotoxic capability of this drug on AML cells, increasing the type 3 DNA toxicity more than twofold (59% vs. 23% with clofarabine alone). Conversely, in Vero cells, the addition of F2 to clofarabine effectively protected the integrity of the genetic material, with cells presenting only the lowest levels of damage (types 1 and 2) and no detectable type 3 genome damage (compared with 9.6% with clofarabine alone) (Figure 4).

### 2.6. Annexin V/SYTOX Green Assay and Multicaspase Activation

**Figure 5:**
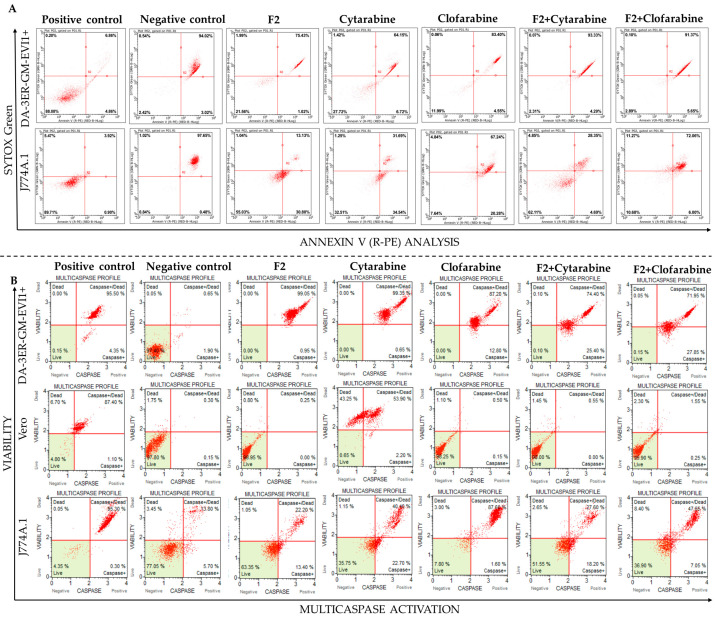
Flow cytometry analysis. (**A**). Annexin V/SYTOX Green; (**B**). Multicaspases activation. F2: citral-rich fraction; positive control: cells treated with DMSO (for Annexin V/SYTOX Green) or Carbonyl cyanide 3-chlorophenylhydrazone (for multicaspases); negative control: untreated cells. Analysis acquired from 5000 events.

Annexin V/SYTOX Green assays revealed PS externalization in 93% of AML cells treated with F2-cytarabine at 48 h, persisting until 72 h. This was significantly higher than the 75% PS labeling observed in cells treated with cytarabine alone, demonstrating that the addition of F2 enhanced its apoptotic effect while maintaining minimal levels of necrosis (0.46% with cytarabine vs. 0.09% mixture) (Figure 5A). Over 99% of AML cells consistently exhibited caspase activation, with 75% undergoing cell death following this activation (Figure 5B).

The cytoprotective effect of the citral-rich fraction F2 on non-tumor cells was confirmed. In Annexin V/SYTOX Green assays, Vero cells treated with F2-cytarabine doubled their viability (24.3% with cytarabine vs. 58.5% with the mixture), and J774A.1 cells increased their viability from 35.8% to 51.5% (Figure 5A). In these macrophages, the cell death phenotype was characterized by PS externalization in 40% of the cells (indicating apoptosis), with only 2% showing necrosis. However, the death of these macrophages associated with caspase activation was significantly reduced when F2 was added to the drug (27.6% in the mixture vs. 99% with cytarabine alone) (Figure 5B).

Annexin V/SYTOX Green assays in tumor cells (DA-3ER/GM/EVI1+) showed that F2-clofarabine therapy induced predominantly apoptotic cell death, reaching 99% death at 72 h, with caspase activation (Figure 5A). This was superior to the 90% PS externalization and 87% death associated with caspase activation observed in AML cells treated with clofarabine alone while maintaining the same necrosis values (0.34%) (Figure 5). In J774A.1 macrophages, the combined therapy showed improved viability rates, increasing from 3.6% with individual treatment to 7% after the combined therapy at 72 h. The death phenotype in these cells was characterized by PS externalization in 85% of the cells, with 8% showing signs of necrosis (Figure 5A), reaffirming that these cells are more sensitive to the treatment, as shown in Figure 2. In contrast, in Vero cells treated with F2-clofarabine, caspase activation was observed in only 2%, like the individual treatment with clofarabine, with a slight increase of necrosis (0.15% with clofarabine vs. 2.3% with the combination) (Figure 5A).

### 2.7. Antioxidant Systems

**Figure 6:**
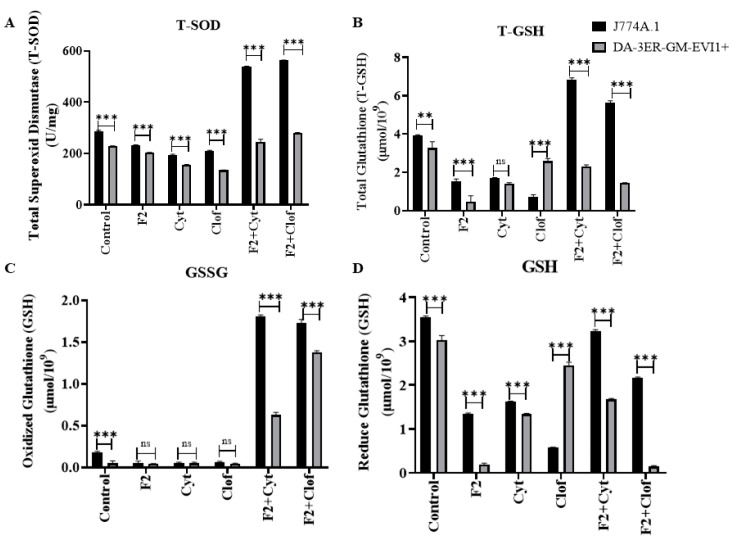
Antioxidant systems. SD: standard deviation; SOD-T: total superoxide dismutase; GSH-T: total glutathione (GSH); GSSG: oxidized glutathione; GSH: reduced GSH; ns: not significant; F2: citral-rich fraction; Cyt: cytarabine; Clof: clofarabine; **: *p* < 0.001; ***: *p* < 0.0001.

The addition of F2 to cytarabine induced a significant increase in antioxidant defense systems in macrophage cells: total superoxide dismutase or SOD-T (196 U/mL for cytarabine vs. 537 U/mL for the mixture), total glutathione or GSH-T (2.3 µmol/10^9^, cytarabine vs. 6.8 µmol/10^9^ for the mixture), and reduced glutathione or GSH (1.6 µmol/10^9^ for cytarabine vs. 3.2 µmol/10^9^ for the mixture) (Figure 6). However, this antioxidant effect of the combined therapy (cytarabine-F2) was not selective and was also observed in leukemic cells (SOD-T: 153 U/mL with cytarabine vs. 353 U/mL with the mixture; GSH-T: 1.4 µmol/10^9^ with cytarabine vs. 2.3 µmol/10^9^ with the mixture; GSH: 1.3 µmol/10^9^ with cytarabine vs. 1.7 µmol/10^9^ with the mixture).

In contrast, the addition of F2 to clofarabine specifically induced oxidative stress in AML cells. Thus, the combined therapy significantly impaired the antioxidant systems [GSH-T (2.6 µmol/10^9^ with clofarabine vs. 1.4 µmol/10^9^ with the mixture) and GSH (2.5 µmol/10^9^ with clofarabine vs. 0.2 µmol/10^9^ with the mixture)], while increasing the pro-oxidant oxidized glutathione (GSSG) levels up to 28 times (0.05 µmol/10^9^ with clofarabine and untreated control vs. 1.4 µmol/10^9^ with the mixture). Interestingly, the pro-oxidant activity of F2-clofarabine therapy was exclusive to tumoral cells. Thus, this mixture significantly potentiated antioxidant defenses in healthy J774A.1 macrophages: SOD-T (211 U/mL with clofarabine vs. 562 U/mL with the mixture), GSH-T (0.7 µmol/10^9^ with clofarabine vs. 5.6 µmol/10^9^ with the mixture), and GSH (0.6 µmol/10^9^ with clofarabine vs. 2.1 µmol/10^9^ with the mixture) (Figure 6).

## 3. Discussion

Conventional treatment of leukemias relies on monotherapies or combined chemotherapies using cytotoxic agents such as cytarabine and clofarabine. Currently, clinical applications of these drugs face significant challenges due to high rates of cancer resistance, particularly in AML. This resistance is characterized by patients surviving 28 days after therapy initiation but either failing to achieve complete remission or experiencing relapse within 1 year of achieving it [3,4]. Notably, after induction therapy with the “7 + 3” regimen, there have been reports of elevated relapse rates, ranging from 50% for patients under 60 years old to 80–90%, for older individuals [21]. Moreover, chemotherapy administration raises critical concerns related to severe toxic side effects, including fatigue, neuropathies, and organic dysfunction in the gastrointestinal, respiratory, and cardiac systems [4,22]. Additionally, these chemotherapeutic compounds exhibit low permeability and penetration into cell membranes, necessitating the use of high-dose regimens associated with severe toxicity and relapses [8]. In pursuing more efficient and less toxic cancer therapies, combining two or more agents with diverse pharmacological (co-adjuvant) properties has become a significant strategy [23,24,25,26]. Combining natural compounds, such as terpenes derived from medicinal plants, with oncological drugs could enhance their permeability and cytotoxic effects on neoplastic cells, allowing for lower doses and reducing nonspecific toxicity [23,24]. Furthermore, due to their complex composition, EO can potentially enhance the antiproliferative effects of chemotherapies through multi-target effects on cellular pathways. These targets include metabolites, receptors, enzymes, ion channels, transporters, nucleic acids, ribosomes, and proteins [27]. Combining therapies with distinct properties in cancer, such as the CHOP-R regimen, has extensively demonstrated excellent benefits, resulting in increased complete remission rates and five-year overall survival [28]. Likewise, a clinical study showed an improved response rate after 12 weeks of treatment in patients with advanced and metastatic breast cancer who received a curcumin regimen in combination with paclitaxel, compared with paclitaxel monotherapy [29].

This study explored complementary therapies based on *L. alba* EO and its citral-rich fraction as potential complementary agents to cytarabine and clofarabine chemotherapies against AML. In leukemia cells, these drugs exhibited superior antiproliferative performance (IC_50_ ranging from 0.63 to 1.1 µg/mL) compared with EO or the fraction. However, these conventional drugs were also highly toxic against non-tumoral cells (CC_50_ ranging from 0.1 to 19.7 µg/mL). Regarding the inhibitory effect of the studied phytotherapy on tumoral cells, good activity was achieved by a fraction derived from *L. alba* EO enriched in citral (F2), with IC_50_ values of 14.7 µg/mL. Notably, lower IC_50_ values (*p* > 0.05) were reported for the F2 fraction compared with those observed from whole oils isolated from the citral chemotype (IC_50_ > 20 µg/mL for EOCit 1 to 5) (Figure 1).

When F2 is combined with chemotherapeutic agents, this compound potentiates these medicines’ selective inhibitory effect on leukemia cells (Figure 2). Thus, in DA-3ER/GM/EVI1+ (AML) cells, F2 demonstrated an obvious synergistic behavior in combined therapy with cytarabine (∑FIC = 0.49 ± 0.15) and clofarabine (∑FIC = 0.54 ± 0.08), enhancing their anti-tumoral effects by eight times. Considering the low permeability of these medicines (primarily cytarabine) through the membrane (low lipophilicity), these synergistic interactions could be attributed to a possible increase in drug permeability at the cell membrane level [30]. Thus, the lipophilic nature and low molecular weight of citral could contribute to increasing the membrane permeability for chemotherapeutic agents [26]. These effects might also be responsible for increased mitochondrial membrane permeability and subsequent loss of its potential, leading to the release of cytochrome c into the cytosol. Coherently, in DA-3ER/GM/EVI1+ cells, the combined therapy of chemotherapeutics with F2 potentiated the ability of the drugs to induce PCD mechanisms rather than necrosis. This shift in the cytotoxic mechanism of chemotherapeutics on tumoral cells depended on oxidative stress induction by increasing mitochondrial ROS, particularly mitochondrial superoxide. This observation was supported by the intensification of MitoSOX^TM^ Red levels and the depletion of mitochondrial membrane potential (Figure 3). In this regard, Navarra et al. (2015) also observed that the interaction induced by adding citral (from bergamot EO) to doxorubicin therapy caused an increase in ROS levels, leading to alterations in mitochondrial membrane potential and the release of cytochrome c, as well as the activation of tumor suppressor genes and PCD pathways in SH-SY5Y human neuroblastoma cells [31]. Similarly, Fang et al. (2017) described how citral increased cytoplasmic ROS activity by pirarubicin, potentiating the in vivo inhibitory effects on colorectal cancer cells [26].

In this study, chemotherapies were the most cytotoxic treatments on non-tumoral cells (Vero and J774A.1). The CC_50_ values for Vero cells ranged from 0.1 µg/mL (cytarabine) to 4.7 µg/mL (clofarabine). For murine macrophages, they ranged from 12.9 µg/mL (cytarabine) to 19.7 µg/mL (clofarabine). Thus, cytarabine was the least selective compound (SI: 0.09). In contrast, F2 demonstrated a significantly lower cytotoxic effect on non-tumoral cells, especially on Vero cells (CC_50_ = 74 µg/mL), while J774A.1 cells were considerably more susceptible to its inhibitory growth cell effect (CC_50_ = 35 µg/mL). However, combined with the studied chemotherapeutics, this fraction demonstrated a cytoprotective effect on non-tumoral cells, consistent with the results from inhibitory growth cell and morphology assays. These analyses revealed that the addition of F2 to both cytarabine and clofarabine restored cell membrane, DNA, and mitochondrial membrane potential integrity while controlling oxidative stress (Figure 3).

Considering the results, the potential pro-oxidant effect of both F2-cytarabine and F2-clofarabine was assessed through antioxidant defense system assays. These analyses revealed that adding F2 to cytarabine had an antioxidant effect, as evidenced by increased SOD-T, GSH-T, and GSH levels in leukemic cells and murine macrophages. Notably, this non-selective antioxidant activity may undermine the antitumor efficacy of chemotherapy, such as simultaneous antioxidant activity, which has been linked to resistance and deleterious effectiveness of antitumor treatments [32].

Conversely, the pro-oxidant effect observed by the MitoSOX^TM^ Red assay when combining F2 with clofarabine was consistent with the results obtained from the antioxidant systems analysis. Specifically, in AML cells treated with F2-clofarabine, there was a significant depletion of antioxidant GSH-T and GSH, accompanied by a strong increase in GSSG. Remarkably, this combined therapy demonstrated dual behavior, acting as a selective antioxidant in J774A.1 macrophage cells. These results align with those previously described by Oien et al. (2016), who found that the selective antiproliferative effect of citral against tumor cells was based on its capability to trigger selective oxidative stress, leveraging the specific vulnerability of these cells to pro-oxidant agents [33]. This susceptibility is due to their elevated levels of basal ROS, associated with their high metabolic rate, excessive proliferation, deficient antioxidant defenses, and mitochondrial dysfunction [20]. Therefore, the overproduction of these free radicals leads to a loss of homeostasis, causing tumor cells to reach their oxidative stress threshold earlier than healthy cells, ultimately inducing selective cell death [34]. Thus, combination regimens involving dual pro-oxidant/antioxidant agents, such as citral, can exhibit cytoprotective (antioxidant) effects on healthy cells. This property can mitigate the side effects of chemotherapy with clofarabine, providing a protective advantage to normal cells.

The citral-rich fraction isolated from *L. alba* EO (F2) also potentiated the genotoxic effect of chemotherapeutic agents on DA-3ER/GM/EVI1+ (Figure 4), while enhancing their selective performance (protecting non-tumoral cells). The combined chemotherapies with F2 also favored an apoptotic phenotype in tumoral cells. These included cytoplasmic blistering, cell shrinkage, nuclear chromatin condensation and fragmentation (confirmed by TUNEL-positive assays), apoptotic body formation, and caspase activation (confirmed by flow cytometry) (Figure 3, Figure 4 and Figure 5). This shift in the AML cell death phenotype could have a favorable effect on alleviating the unwanted side effects of chemotherapy, especially those related to necrotic cell death, such as the severe inflammatory response in normal organs.

Similar apoptotic effects were reported by Garcia et al. (2017) in K562 cells treated with EO isolated from *L. alba* citral chemotype, composed of high percentages of citral (ranging from 50 to 85%) [11]. The antitumoral action of citral mediated by PCD mechanisms was also described in mammary, gastric, hepatic, ovarian, and hematological carcinogenic cells [13,35,36,37]. This activity was attributed to the caspase-3 activator effect of the α, β-unsaturated chemical group present in citral [38], as well as an increase in Bax and a reduction in Bcl-2 and NF-κB, resulting in the loss of mitochondrial membrane potential [37]. In this regard, a synergistic antiproliferative and selective activity of the citral-doxorubicin mixture was observed on Burkitt’s lymphoma cells, which was associated with an intensification of the apoptotic stimulus (elevated levels of the pro-apoptotic protein Bak and diminished expression of the anti-apoptotic agent Bcl-xL) [25].

Combined therapies of conventional chemotherapeutics and natural compounds, like F2, promise to improve leukemia treatments. These therapies can enhance the efficacy of chemotherapeutic drugs while minimizing non-specific cytotoxicity. The observed effects of the combined therapies on tumor cells were attributed to altered mitochondrial function, oxidative stress induction, and activation of PCD pathways. These findings contribute to the ongoing efforts to develop more efficient and less toxic therapies against AML.

## 4. Materials and Methods

### 4.1. Cell Cultures

Selectivity index assays were assessed on Vero (ATCC-CCL 81) and J774A.1 (ATCC TIB-67) cells, cultured in Dulbecco’s Modified Eagle Medium (DMEM) (Gibco, CA, USA), pH 7.2, supplemented with 10% inactive Fetal Bovine Fetal Serum (iFBS) (Gibco, Carlsbad, CA, USA), 1000 U/mL penicillin, and 100 µg/mL streptomycin (Gibco, Carlsbad, CA, USA). Cytotoxicity assays were performed on AML [DA-3ER/GM/EVI1+] [39] cultured in Roswell Park Memorial Institute medium (RPMI-1640) (Gibco, Carlsbad, CA, USA) and supplemented under the same conditions described above for non-tumor cells. All cells were grown at 37 °C with 95% humidity and 5% CO_2_. [DA-3ER/GM/EVI1+] cells are myeloid progenitors, with EVI1 oncogene [ecotropic virus insertion site 1] overexpression [39], which were modified for autonomous growth by the autologous, non-secretable expression of Monocytic Granulocytic Colony Stimulating Factor (GM-CSF) directed to the endoplasmic reticulum [40].

### 4.2. Chemotherapeutics

Cytarabine (50 mg/mL) and clofarabine (1 mg/mL) were kindly donated by Empresa Social del Estado—Hospital Universitario de Santander (ESE-HUS). The working solutions (100 µg/mL for both chemotherapeutics) were diluted in DMEM (Gibco, Carlsbad, CA, USA) or RPMI-1640 (Gibco, Carlsbad, CA, USA) for the assays on non-tumoral and tumoral cells, respectively.

### 4.3. Plant Material

The citral chemotypes of *L. alba* (Mill) N. E. Brown (Verbenaceae) plants were grown in the National Research Center for Agroindustrialization of Aromatic Medical and Tropical species (CENIVAM, in Spanish), in Bucaramanga (Santander), Colombia, at 7°8′26″ N 73°7′10″ W, at 960 m above sea level altitude, under standardized environmental and production conditions [11]. Prof. Jorge Luis Fernández Alonso officially identified the plant specimens using the conventional taxonomic approach described by Moldenke (1971) [41]. This identification was based on shared superficial morphological characteristics of the plant (leaves, fruits, inflorescences, stems, and branches) by comparison with previously collected samples and identification guides. The vouchers were deposited at the Colombian National Herbarium (Universidad Nacional de Colombia) under Herbarium Code COL512077.

### 4.4. Isolation and Chemical Characterization of Essential Oils and Fraction

EO were extracted from 100 g of mature and young leaves in 0.5 L of water, which were submitted to distillation by microwave-assisted hydrodistillation (MWHD) technique as previously described [11]. Alternatively, the fraction (F2) isolation procedure was performed from EO extracted by steam distillation technique and then subjected to reduced-pressure fractional distillation in a B/R Instruments (Easton, MD, USA) 800 High-Efficiency Micro Distillation device, as described in [16].

### 4.5. Cytotoxic Activity on Vero and J774A.1 Cells

Vero (3 × 10^4^ cells/mL) and J774A.1 (1.5 × 10^4^ cells/mL) were incubated in 96-well flat-bottom plates at 37 °C, 5% CO_2_, and 95% humidity for 24 h until a confluent monolayer was formed. Then, the cells were treated with different concentrations of the therapies (from 11.1 to 300 μg/mL for essential oils and the fraction; or from 0.04 to 5 μg/mL for chemotherapeutics) and incubated under the same conditions described above for 24 h. After that, the cell proliferation reagent WST-1 (Roche Applied Sciences, Mannheim, Germany) was added, and cells were re-incubated for 2 h, after which the optical density (OD) was determined spectrophotometrically at a wavelength of 450 nm using a Multiskan Sky Microplate Absorbance Reader (Thermo Scientific, Waltham, MA, USA). The percentage inhibition was calculated as described previously [16]. The results were reported as CC_50_.

### 4.6. Antiproliferative Activity on AML Cells

DA-3ER/GM/EVI1+ cells (3 × 10^4^ cells/mL) were seeded in 96-well flat-bottom plates and incubated for two hours at 37 °C, 5% CO_2_, and 95% humidity. Subsequently, the different concentrations of EO or the enriched fraction (3.7 to 150 μg/mL) and chemotherapeutics (0.02 to 22.5 μg/mL) were added and incubated under the same conditions for 24 h. The cell proliferation reagent WST-1 (Roche Applied Sciences, Mannheim, Germany) was used to determine cell inhibition, as described above. The results were reported as IC_50_.

### 4.7. Pharmacological Interaction between F2 Fraction and Chemotherapeutics

A matrix of pharmacological interactions between the F2 fraction and the reference chemotherapeutics was performed on DA-3ER/GM/EVI1+ cells, using the matrix suggested by Fivelman et al. (2004) [42]. In the matrix, the IC_50_ values determined for F2, and chemotherapeutics were used as fixed values for the interactions (Table 2). Then, the inhibitory percentage was evaluated on Vero and J774A.1 cells. The susceptibility assessment was carried out following the protocol above-mentioned for in vitro antiproliferative activity. The results were expressed using the fixed isobologram method [43]. Fractional Inhibitory Concentration (FIC) was calculated to evaluate susceptibility using Equation (1):

FIC = (compound IC_50_ in combination)/(compound IC_50_ alone). (1)

The sum of FIC (ΣFIC) was defined in Equation (2)

ΣFIC = (FIC F2) + (FIC chemotherapy)(2)

In this manner, synergistic: $\bar{X}$ΣFIC < 1; antagonistic: $\bar{X}$ΣFIC > 1; and additive: $\bar{X}$ΣFIC = 1 interactions were defined [43].

### 4.8. Cell Death Analysis

Cell death tracking was performed by optical and fluorescence microscopy (Fluorescence microscopy, Nikon Eclipse Ni, Tokyo, Japan) on 4 × 10^4^ cells/mL of Vero and DA-3ER/GM/EVI1+ cells seeded in 96-well flat-bottom plates. The cells were incubated under the same conditions as before mentioned for antiproliferative assays. Morphological changes triggered by the most synergic mixtures between the fraction and chemotherapeutic agents (2X IC_50_ F2 + IC_50_ chemotherapy) were analyzed using Giemsa dye (IHR Diagnostic, COL). DNA fragmentation was examined using a TUNEL assay (Molecular Probes, Invitrogen, CA, USA). For mitochondrial membrane potential (ΔΨm), cells were stained with 5 μM of MitoProbe™ JC-1 Assay (Life Technologies, Carlsbad, CA, USA) and incubated at 37 °C for 20 min (529 nm/590 nm excitation/emission wavelength). Superoxide production was evaluated using 4 μM of MitoSOX™ Red (Life Technologies, Carlsbad, CA, USA) and incubating the cells for 30 min at 37°C (510 nm/580 nm excitation/emission wavelength). Controls were provided by untreated cells (negative) and by cultures treated with Carbonyl Cyanide 3-chlorophenylhydrazone (CCCP) (positive) by the manufacturer’s instruction.

A Muse Multicaspase (Cytek) kit and an Annexin V/Dead with SYTOX Green (Molecular probes, Invitrogen, Waltham, MA, USA) kit were used to determine the cell death phenotype, following the manufacturer’s instructions. Briefly, cells treated for 48 h with the different studied therapies (individual and combined) were washed and suspended in 1X caspase buffer. Then, Muse Multicaspase Reagent Working was added and incubated at 37 °C, 5% CO_2_, and 95% humidity for 30 min. After incubation, Muse Caspase 7-Aminoactinomycin D (7-AAD) Working Solution was incubated at room temperature for 5 min. Cells were analyzed using a Muse Flow Cytometer (Merck, Darmstadt, Germany).

PS externalization was determined using a recombinant Annexin V conjugated to the Orange Fluorescent phycobiliprotein R-PE and SYTOX™ Green nucleic acid stain for necrotic cells. Cells treated for 48 h were suspended in 1X Annexin-Binding Buffer. Next, R-PE Annexin V and SYTOX^®^ Green Stain were added and incubated at 37 °C, 5% CO_2_, and 95% humidity. Finally, the samples were analyzed using a Guava easyCyte Flow Cytometer (Merck, Darmstadt, Germany).

### 4.9. Effect on Antioxidant Systems

J774A.1 and DA-3ER/GM/EVI1+ cells were arranged in 12-well plates and exposed for 24 h to the different compounds (IC_50_ of individual and 2X IC_50_ F2 + IC_50_ chemotherapy of combined therapies). SOD-T activity was determined using a T-SOD Activity Assay Kit (MyBioSource, CA, USA); and total (GSH-T), reduced (GSH), and oxidized (GSSG) Glutathione by a GSH-T Kit (MyBioSource, San Diego, CA, USA). All determinations were made following the manufacturer’s instructions for sonicated cells.

### 4.10. Genomic Damage

Vero and tumoral (3 × 10^4^ cells/mL) cells were treated for 24 h with F2 or chemotherapeutics (CC_50_ or IC_50_, respectively). The best pharmacological interactions (2X IC_50_ F2 + IC_50_ chemotherapy) were centrifuged, and the pellet was mixed with low-melting-point agarose (0.5%) to be added to slides previously treated with agarose 1.5%. After that, those slides were immersed in lysis buffer for 2 h and washed with alkaline buffer for 15 min. The electrophoretic run was carried out at 25 V for 40 min. Once the run was over, slides were immersed in a neutralization buffer and stained with DAPI (1 µg/mL, Sigma Aldrich, St. Louis, MO, USA). DNA migration as an indicator of damage to the genome was determined by visual scoring, considering the length of the comet’s tail as previously described [44].

### 4.11. Data Analysis

Xlfit™ 5.5.0.5 statistical software (IDBS, Boston, MA, USA) was used to calculate CC_50_ and IC_50_ by sigmoidal regression and to analyze the pharmacological interactions. To determine the statistically significant differences in the genotoxicity test, growth inhibition, and measurement of antioxidant systems, an ANOVA test was conducted in GraphPad Prism 9.5.1 (GraphPad Software Inc., Boston, MA, USA). Dunnett’s test performed multiple comparison analyses at a 95% confidence level. These experiments were assessed in triplicate in three independent trials, and measurement data were expressed as the mean ± standard deviation. This study was approved by the ethics committees of Universidad de Santander and Universidad Industrial de Santander, Agreement 010-VII May 15 and 16, 2017.

## Figures and Tables

**Table 1:** Relative chemical composition and linear retention indices (LRI) by GC-MS of *L. alba* essential oils (EO) and its citral-rich fraction (F2).

Compound	LRI		Relative Quantity (%)	
	DB-5MS	DB-WAX	Citral Chemotype	
			EOCit1–5	F2
6-Methyl-5-hepten-2-one	986	1241	3.3	-
Limonene	1034	1105	6.6	-
Linalool	1100	1453	1.9	0.2
Citronellal	1154	1381	1.1	-
Nerol	1231	1708	0.8	2.0
**Neral**	1248	1589	**21.5**	**31.5**
Geraniol	1252	1755	5.6	7.9
**Geranial**	1275	1643	**28.7**	**40.1**
Geranyl Acetate	1379	1662	1.5	-
β-Elemene	1397	1496	3.0	-
trans-β-Caryophyllene	1436	1506	12.1	9.8
α-Guaiene	1447	1498	1.8	1.6
α-Humulene	1471	1580	2.7	1.2
Germacrene D	1486	1552	2.6	0.4
Bicyclosesquiphellandrene	1496	1624	-	0.3
α-Bulnesene	1515	1627	1.4	-
Caryophyllene oxide	1600	1909	2.3	1.0

DB-5MS: LRI experimentally determined in DB-5MS (60 m) column; DB-WAX: LRI experimentally determined in DB-WAX (60 m) column; EOCit1-EOCit5: EO from citral chemotype of *L. alba*. The compounds in bold are the terpenes of greatest interest, as they constitute the majority of the essential oil and the enriched fraction.

**Table 2:** Interaction matrix.

Combination ID Number	F2		Cytarabine/Clofarabine	
	(IC_50_)	(μg/mL)	(IC_50_)	(μg/mL)
1	0.0	0.0	8X	8.8/5.0
2	½X	7.4	4X	4.4/2.5
3	1X	14.7	2X	2.2/1.3
**4**	**2X**	**29.4**	**1X**	**1.1/0.63**
5	4X	58.8	½X	0.55/0.32
6	8X	117.6	0.0	0.0/0.0

IC_50_: Inhibitory Concentration 50; X: number of times; the most synergistic mixture is in bold.

## Data Availability

The necessary data can be requested from the correspondence author.
